# The Refining of Crude Statistics

**Published:** 1916-11-11

**Authors:** 


### ENGLAND'S RECENT PROGRESS.

### The Refining of Crude Statistics.

Progress is a word of many implications, but we take it that Mr. Welton* uses it in a strictly statistical sense. Even so limited it has a positive and a negative significance: like the mathematician's "acceleration," it may be a speeding up or a slackening down. Change in the distribution of population within defined areas is the subject to which Mr. Welton has devoted himself for years, and this large volume records the results of his analysis of the last Census returns.

Books such as this, composed largely of tables and appendices, do not make light reading. They are apt to lie heavily on the shelves, to be looked into to-morrow or next week, or they may be taken down occasionally in search of figures to support an argument—which in the view of an eminent statesman is the principal use of statistics. Certainly many foregone conclusions are supported by Mr. Welton's figures. He shows us the slackening of the birth-rate within two successive periods of ten years; but he also shows us that the rate of slackening is different in different portions of the community, being greatest in residential districts and least in mining districts. He invites us generally to regard the Registrar-General's figures as what they really are, an arithmetical mean of a vast number of groups. A general increase in population may cover an actual decrease in certain agricultural districts at the same time as a vast increase in certain attractive localities.

The Study of Localities.

The study of typical localities has been Mr. Welton's object for some years. He has contributed an interesting and important study of the "Industrial Changes of Forty Years" to the Manchester Statistical Society, and a paper on the distribution of population in the ninety years, 1801-91, to the journal of the Statistical Society. It would have been a great advantage if he had reprinted these two papers as part of the present volume.

Still better would it be if Mr. Welton could give us a statistical history of half-a-dozen specimen communities, taking for instance such a town as Coventry, which has had successive cycles of depression and prosperity, or such a community as Bournemouth, which within a period of thirty years from a small seaside resort advanced to the proportions of a large town. Such a study would be really more enlightening than any of the national averages which the Registrar-General gives us.

When we visit any town for the first time we are tempted to ask not What is your exact population? but How are your people maintained, what are they producing, or are they perchance not producing at all but living on accumulated means, like bees in the early spring on last year's honey? Mr. Welton would probably say, and with truth, that the character of the most attractive places—the places showing the greatest recent increase of population—affords an important clue to the general condition of the country. If you see a health resort showing an increase of population far exceeding the general rate, with hundreds of new houses of a stately type filling as fast as they are built, the conclusion is irresistible that the country of which it forms a part is as a whole prosperous. The migrants into such a place represent for the most part successful industry either of the present or of the immediate past. If you desired to show to a foreigner some evidence that England is still a wealth-producing country you would produce more impression upon him if, instead of taking him to a manufacturing town and showing him the machinery in action, you took him to some such favoured residential district and showed him the resorts in which the people enjoyed their leisure.

On the two prominent topics the decline in the birth-rate and the depletion of agricultural areas, Mr. Welton gives figures showing considerable variations in different districts. In the colliery districts the reduction in the birth-rate has been very slight; it has been greatest in residential districts and in textile manufacturing places. Mr. Welton takes as the standard of natural fertility 3,200 births per 10,000 married women aged under forty-five. This standard is still very nearly attained in colliery and industrial districts, but the decline in other portions of the community is greater than that shown in the Registrar-General's averages.

The Celibate North.

As to the general marriage rate Mr. Welton concludes that there is a greater tendency towards marriage in the mainly agricultural Eastern and Western counties than in the mainly industrial Northern counties, and that throughout the country the marriage rate is higher in towns than in rural districts. An average marriage rate of 14.6 per 100 unmarried men aged twenty to thirty-five conceals the fact that in the towns of Stafford and Leicester the rate was 15.1, whereas in the rural parts of the same counties the rate was only 12.6. As regards the low marriage rate of the Northern counties Mr. Welton suggests that the higher wages paid in the North have led to a more expensive standard of living and an indisposition to marry if that standard would be imperilled.

The next Census should be the most momentous since these decennial returns were first instituted. Some effort should be made to obtain special returns for those districts which have been specially affected by military preparations, by munition work and the like. Valuable annual reports are made in many towns by the medical officer, and the salient features of these should be made accessible to the public either as an appendix to the Census return or otherwise. There are many problems of population, of production, of occupation, on which existing conditions are throwing a flash of light. One would not have supposed that our existing produc-

* *England's Recent Progress.* By Thomas A. Welton, F.S.S., F.C.A. London: Chapman and Hall. Price 10s. 6d. net.

tion is only about one-third that of which our machinery is capable, yet that is evidently the fact. Few would have supposed that two-thirds of the clerical and office work hitherto done in this country is absolutely wasted and could be dispensed with. Events are demonstrating that also, though the demonstration has not yet had time to penetrate very far. It is remarkable that our War Office and Ministry of Munitions has a staff five times as large as that which efficiently controls the corresponding offices in France. The next Census of production and the next Census of population ought to be the most instructive documents yet published on the Condition of England.

